# Altering Host Resistance to Infections through Microbial Transplantation

**DOI:** 10.1371/journal.pone.0026988

**Published:** 2011-10-28

**Authors:** Benjamin P. Willing, Anjalee Vacharaksa, Matthew Croxen, Teerawat Thanachayanont, B. Brett Finlay

**Affiliations:** 1 Michael Smith Laboratories, The University of British Columbia, Vancouver, British Columbia, Canada; 2 Department of Microbiology and Immunology, The University of British Columbia, Vancouver, British Columbia, Canada; Charité-University Medicine Berlin, Germany

## Abstract

Host resistance to bacterial infections is thought to be dictated by host genetic factors. Infections by the natural murine enteric pathogen *Citrobacter rodentium* (used as a model of human enteropathogenic and enterohaemorrhagic *E. coli* infections) vary between mice strains, from mild self-resolving colonization in NIH Swiss mice to lethality in C3H/HeJ mice. However, no clear genetic component had been shown to be responsible for the differences observed with *C. rodentium* infections. Because the intestinal microbiota is important in regulating resistance to infection, and microbial composition is dependent on host genotype, it was tested whether variations in microbial composition between mouse strains contributed to differences in “host” susceptibility by transferring the microbiota of resistant mice to lethally susceptible mice prior to infection. Successful transfer of the microbiota from resistant to susceptible mice resulted in delayed pathogen colonization and mortality. Delayed mortality was associated with increased IL-22 mediated innate defense including antimicrobial peptides Reg3γ and Reg3β, and immunono-neutralization of IL-22 abrogated the beneficial effect of microbiota transfer. Conversely, depletion of the native microbiota in resistant mice by antibiotics and transfer of the susceptible mouse microbiota resulted in reduced innate defenses and greater pathology upon infection. This work demonstrates the importance of the microbiota and how it regulates mucosal immunity, providing an important factor in susceptibility to enteric infection. Transfer of resistance through microbial transplantation (bacteriotherapy) provides additional mechanisms to alter “host” resistance, and a novel means to alter enteric infection and to study host-pathogen interactions.

## Introduction

It is well known that there are varying levels of individual susceptibility in a mixed population to infectious agents, and several host resistance factors have been identified in both mice and humans that contribute to susceptibility [1]. For example, a single genetic locus in the mouse, *nramp1/SLC11A1*, confers susceptibility and resistance to murine models of typhoid, tuberculosis, and leishmaniasis [1]. However, there are several examples where strain variability is well known, yet few or no host genetic factors have been identified.

Infections of food-borne pathogens, such as enteropathogenic *Escherichia coli* (EPEC) and enterohemorrhagic *E. coli* (EHEC), cause a disturbance in the microbial niche followed by gastrointestinal (GI) inflammation and sometimes life-threatening diarrhea [2]. Similar to diseases in humans, *Citrobacter rodentium* colonizes the GI tract of mice and induces the same characteristic attaching and effacing (A/E) lesions and mucosal inflammation in mouse models [3]. Severity of *C. rodentium*-induced infections, including inflammation, has been shown to vary among mice of different genetic backgrounds [3], [4]. Self-limited disease with low or no mortality frequently occurs after *C. rodentium* infection in many strains of mice including NIH Swiss (NIH) and C57Bl/6, but for C3H/HeJ (HeJ) [3] and most FVB/N mice [5], the infection is lethal.

Adaptive immunity is needed for the control and clearance of *C. rodentium* [6] *;* however, innate mucosal defenses including mucin expression and antimicrobial peptides are important for its epithelial attachment and luminal colonization [7], [8]. Despite the fact that C3H/HeJ mice appear to have functional innate mechanisms, it remains unclear why they are quickly overcome by this pathogen.

Recently, it has become apparent that the microbiota plays a key role in affecting the outcome of infection, as treatment with antibiotics or other factors that affect microbiota can change the outcome of infection to various pathogens in an individual host strain [9], [10]. Studies have revealed that the mucosal immune system is dynamic depending on continued microbial signaling [11]–[13]. Gastrointestinal (GI) microbiota maintain equilibrium at the mucosa by preventing pathogen adherence through the induction of host innate immune defenses [11], [14]. For example, gut commensals have been shown to mediate IL-22 expression, a cytokine important for controlling *C. rodentium*-induced enterocolitis in C57Bl/6 mice [15]. Segmented filamentous bacterium (SFB) colonization in the distal ileum has been associated with the homeostasis of CD4+ T helper cells in the lamina propria, and the induction of cytokines including IL-17 and IL-22 as well as downstream antimicrobial peptides regenerating islet-derived 3 (Reg3)γ and Reg3β [16]. Furthermore, TLR signaling through MyD88-dependent, IL-1R- and IL-22-dependent signaling pathways confines infection to the mucosal surface and prevents bacteremia [8], [17]–[20].

Because genetic background of the host guides gut microbial composition [21] [22] and no clear genetic divergence had been shown to explain the difference in disease severity between different mouse strains infected with *C. rodentium,* we tested the role of different microbiotas in disease resistance using microbiota transplantation between mouse strains. We postulated that GI microbiota specific to HeJ mice lacked essential constituents that could protect the intestinal mucosa from *C. rodentium* colonization and infection. To investigate the role of GI microbiota, we developed a mouse model using microbiota transplantation in non-germfree mice. Using these models, the susceptibility of HeJ mice carrying NIH Swiss mice microbiota (HeJ-NIH) was compared to mice of the same genetic background carrying HeJ microbiota (HeJ-HeJ). Mouse-specific GI microbiota that influenced colonization of A/E bacterial pathogens and host susceptibility was then characterized, and shown to play a critical role in determining the outcome of infection.

## Materials and Methods

### Ethics Statement

All animal experiments were performed in strict accordance with the guidelines of the University of British Columbia Animal Care Committee and the Canadian Council on the Use of Laboratory Animals. The protocol was approved by the UBC Animal Care Committee (Certificate number:A09-0168). The mice were euthanized by CO2 asphyxiation and all efforts were made to minimize suffering.

### Mice and microbiota transplantation

Three-week-old female NIH Swiss mice (Harlan Laboratories, Inc., Indianapolis, IN), and C3H/HeJ mice (Jackson Laboratory, Bar Harbor, ME) were housed at the University of British Columbia. The gut microbiota was transferred between groups of mice by initially depleting the native microbiota with a single oral dose of streptomycin (20 mg) 24 hours prior to transplantation. Fresh fecal pellets from 3–4 donor mice were collected and placed in 1 mL of transfer buffer (pre-reduced sterile phosphate buffered saline containing 0.05% cysteine HCl (Sigma-Aldrich; Missouri, USA)) on ice. The fecal pellets were homogenized, centrifuged at 800× *g* for 2 min and the supernatant was collected and diluted (1∶3) in transfer buffer. One hundred µL of diluted fecal supernatant was then orally inoculated to recipient mice over the subsequent 12 days for a total of 6 times. To control for the effect of the transfer process a control group was always included that received transplantation from fecal pellets of mice of the same strain.

### Microbial community analysis

Fecal samples were collected fresh for determination of microbial composition and stored at -20°C. Total DNA was extracted using the QIAamp DNA stool kit (Qiagen, Mississauga, ON) according to manufacturer's instructions with the addition of a bead-beating step. The bacterial community profile was assessed by terminal restriction fragment length polymorphism (T-RFLP) as previously described [23] using broad-range bacterial primers (Table S1) that amplify variable regions V1 to V5 and restriction digestion with HaeIII and MspI (New England Biosciences, Beverly, MA). Using the same primer sequences as for T-RFLP, cloning and sequencing was used to assess community composition of a subset of samples using TOPO TA 4.0 vector and *E. coli* TOP 10 chemically competent cells (Invitrogen, Carlsbad, CA). Six separate clone libraries (60-64 clones/library) were generated from DNA pooled between mice in a cage for 2 cages for each NIH, HeJ-NIH and HeJ groups. Sequences with an ambiguous base were not included and tested for chimeras and deposited in Genbank (JF837825-JF838122). Quality sequences were aligned with the greengenes reference alignment and clustered at an operational taxonomic unit (OTU) sequence similarity of 97% in the MOTHUR platform [24]. Phylogenetic trees were compared using libshuff in MOTHUR. Classification of sequences was done using the naïve Bayesian rRNA classifier in RDP [25]. Quantification of select bacterial populations was assessed by real-time PCR (Primers in Table S1) on an ABI7000 (Applied Biosystems, Foster City, CA) using SYBR green qPCR mix (Qiagen) and standard curves generated from known isolates.

### C. rodentium infection

Wildtype *C. rodentium,* strain DBS100, was grown overnight in Luria broth (Sigma-Aldrich; Missouri, USA) with shaking. Mice were infected by oral administration of 5×10^8^ (high dose) or 5 × 10^4^ CFU (low dose) by gavage 2 days after the last transfer (d14).

### Host susceptibility assessment and pathogen colonization

Mice were monitored for weight loss and other clinical signs. Sick mice were euthanized when body weight loss was greater than 20% of the initial weight. Surviving mice were presented as a percentage of the initial number. Pathogen load was monitored by plating on MacConkey agar (Difco Laboratories; Michigan, USA).

### Host gene expression

Quantification of gene expression was assessed in ileum samples by real-time PCR (Primers in Table S1) on an ABI7000 (Applied Biosystems, Foster City, CA) using SYBR green qPCR mix (Qiagen).

### Histopathology of gut inflammation and immunohistochemistry

Formalin-fixed tissues were embedded in paraffin and 5 µm sections were taken for histopathological and immunochemistry analysis. Hematoxylin and eosin stained tissues were scored for pathology in four regions including the lumen, surface epithelium, mucosa and submucosa as previously described [26]. For immunohistochemistry rehydrated tissue sections were blocked with 10% normal goat serum in staining buffer (3% BSA, 0.1% Triton X100, 0.05% Tween20) and then incubated with sheep anti-mouse Reg3β (R&D systems) diluted 1∶100 in staining buffer overnight at 4°C. The secondary antibody, goat anti-sheep IgG conjugated with HRP diluted 1∶1000 in staining buffer was applied for 1 h at room temperature. Finally, tissue sections were incubated with DAB substrate (BD) for 20 min, thoroughly rinsed, and hematoxylin counterstained. Slides were mounted with Permount (Fisher) and viewed under light microscope. Pictures were taken on Zeiss Axiocam and the images were obtained using Axiocam software (Skokie, IL).

### IL-22 neutralization

A monoclonal mouse anti-human IL-22 antibody or isotype control at a dose of 100 µg (R&D Systems, Minneapolis, MN) was administered intraperitoneally (IP) to HeJ-NIH and HeJ-HeJ mice for 3 consecutive days beginning 2 days prior to infection and ending on the day of infection. Mice were given the high infection dose and monitored for weight loss as described above. Samples were harvested from a second set of mice after 2 doses of IL-22 and isotype antibodies to determine the effects on Reg3β expression.

### Statistical analyses

The survival curves of infected mice were compared using Kaplan-Meier analysis followed by log-rank test. Bacterial loads and body weights were compared using two-tailed Student's *t*-test. One-way ANOVA was used for comparisons of multiple treatments. All analyses were performed with a 95 or 99% confidence interval using GraphPad Prism software, version 4.0. Multivariate analysis of microbial community composition assed by T-RFLP included non-metric multidimensional scaling (NMS) plotting and cluster analysis using Bray-curtis metrics in R. Significance of treatment was tested by multiple response permutation procedure (MRPP). Mean values in text are presented ± standard error.

## Results

### NIH microbiota colonize C3H/HeJ mice after microbiota transplantation

**Figure 1:**
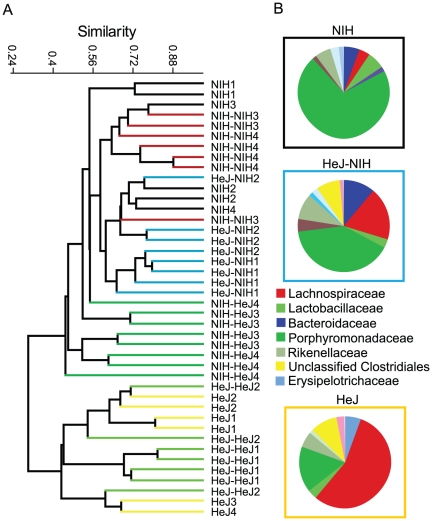
Analysis of microbial 16S rRNA gene composition indicates successful transplantation of microbiotas between mouse strains. (a) Similarity tree using Bray Curtis metrics of bacterial 16S rRNA gene terminal restriction fragment profiles from untreated C3H/HeJ (HeJ) and NIH Swiss (NIH) mice and HeJ or NIH mice having received oral microbial transplantation from HeJ mice (HeJ-HeJ or NIH-HeJ) or NIH mice (HeJ-NIH or NIH-NIH). Mice receiving transplantation were from 4 separate experiments and represent multiple cages in each experiment. The number denotes which experiment the samples were from. (b) Classification of 16S rRNA gene clone libraries at the family level generated from fecal samples from HeJ, NIH and HeJ-NIH mice.

Before we could determine whether transplantation between mouse strains was possible we had to determine whether microbiotas were specific to mouse strain. Indeed the microbiota of NIH and HeJ mice are clearly distinct (Fig. 1a). After pretreatment with streptomycin the transfer of NIH microbiota to HeJ mice over a period of 12 days was quite efficient. The majority of the T-RFLP phylotypes specific to NIH mice prior to transfer appeared in similar abundances in HeJ-NIH mice and the fecal bacterial profiles (d14) of HeJ-NIH mice clustered tightly with NIH mice (Fig. 1a), rather than HeJ. The microbiota of HeJ-HeJ clustered with HeJ-Naïve mice after microbiota transplantation. Bacterial 16S rRNA gene clone libraries of variable regions V1-V5 generated from DNA isolated from the feces (d14) of HeJ-Naïve, NIH and HeJ-NIH provided taxonomic information of transferred bacteria (Fig. 1b and Fig. S1 and S2). Prior to transfer HeJ mice had a low abundance and diversity of *Bacteroidetes* relative to NIH. After transfer there was increased abundance of diverse members of the *Bacteroidetes* phylum, including *Prevotella, Tannerella, Bacteroides, Rikenellaceae* and *Barnsiella.* The abundant representation of *Lachnospiraceae* family in HeJ mice (48-68% of clones) fell substantially in HeJ-NIH mice (17–22% of clones), to more closely resemble the *Lachnospiraceae* population in NIH mice (2–6% of clones). Prior to transfer, NIH and HeJ mice had only 3 OTUs (defined at 97% similarity) in common, whereas after transfer HeJ-NIH mice shared nearly a third (18/61) of the OTUs detected in the clone libraries of NIH mice. While libshuff analysis of phylogenetic trees generated from the respective clone libraries indicated that differences remained in the communities of NIH and HeJ-NIH mice (*P* = 0.01), the HeJ-NIH mice were more similar to NIH mice than they were to HeJ-Naïve mice (*P*<0.0001). HeJ-NIH mice still shared 6 of 71 OTUs with HeJ-Naïve mice, suggesting that the HeJ microbiota was not completely displaced. Quantification of select bacterial groups by real-time PCR confirmed an increase in members of the *Porphyromonadaceae* family and decrease in *Lachnospiraceae* observed in T-RFLP and clone libraries in HeJ-NIH mice (Fig. S3). Segmented filamentous bacteria were below detection limits for T-RFLP in fecal samples; however, qPCR revealed a shift according to mouse-specific microbiota. Relative abundance of SFB was 0.05% and 0.15%, in HeJ-NIH and NIH-specific microbiota, respectively, but SFB were near the limit of detection in HeJ-specific bacterial communities.

### HeJ mice carrying NIH microbiota are less susceptible to *C. rodentium* infections

Once it was established that the microbiota could be transferred between NIH and HeJ mice, it was possible to determine whether the microbiota from resistant mice could confer resistance to susceptible mice. Three independent experiments were performed using high dose infection (5×10^8^ CFU). When mice showed >20% weight loss from the initial body weight measured on day 1 post infection (pi), they were euthanized and the end points were recorded for survival assessment.

**Figure 2:**
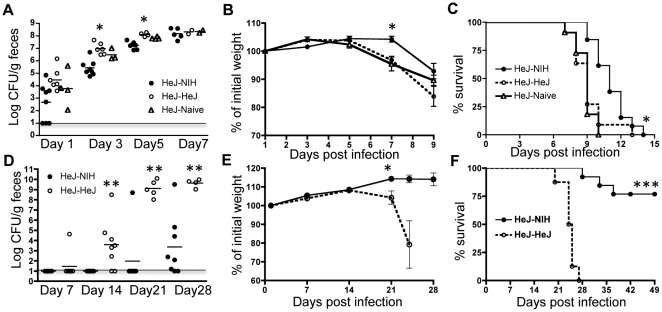
Mice with NIH microbiota showed reduced susceptibility to *C. rodentium* infection. After microbiota transplantation, mice were challenged with 10^8^ or 10^4^ colony forming units (CFU) of *C. rodentium.* Shedding of *C. rodentium,* body weight loss and survival were assessed for host susceptibility. Data is representative of three independent experiments with high infection dose (a,b,c), or two independent experiments with low infection dose (d,e,f). HeJ-NIH mice carrying NIH microbiota had significantly lower *C. rodentium* shedding in feces at the early time points post infection, delayed weight loss and mortality. Weights are represented as mean±SEM. (n = 8 for each experiment).

*C. rodentium* shedding was delayed in HeJ-NIH mice as compared to HeJ-HeJ and HeJ-Naïve mice and significantly lower on day 3 and 5 pi (*P*< 0.001) (Fig. 2a). Consequently, HeJ-NIH mice demonstrated delayed weight loss (Fig. 2b) and mortality (Fig. 2c) (*P<*0.05) after high dose infection (consistent in all 3 experiments). From these results we could conclude that at least partial resistance to *C. rodentium* could be transferred between resistant and susceptible mice by microbial transplantation.

Although HeJ-NIH mice had delayed disease with the high dose infection, they eventually succumbed to infection, suggesting that the microbial transplantation only delayed establishment of the infection. Since the high-dose infection may have overwhelmed the system, we instead challenged mice with a more realistic low infection dose (5×10^4^ CFU) (Fig. 2d,e,f). As expected, low dose infection slowed the kinetics of *C. rodentium* infection in all HeJ mice when compared to the results of high infection dose. After low dose infection, HeJ-HeJ mice showed higher and more rapid colonization of *C. rodentium* than HeJ-NIH mice followed by severe weight loss by approximately 2 weeks and 3 weeks in the first and second challenge, respectively. As a result, euthanization was required for HeJ-HeJ mice while significant numbers of HeJ-NIH mice survived the infection. In the first low-dose infection experiment all mice eventually succumbed to disease, but remarkably, in the second low-dose challenge experiment over 70% of HeJ-NIH mice were able to clear the bacterial infection and survived, yet all HeJ-HeJ succumbed to infection. Interestingly, the mean similarity of the microbiota between HeJ-NIH mice and NIH donors, as measured by Bray-curtis metrics, was slightly higher (68.0±2.1% vs 57.3±3.6% similarity (*P*<0.05)) in the second experiment, although this was not attributable to a single phylotype.

### NIH mice carrying HeJ microbiota are more susceptible to *C. rodentium* infection

**Figure 3:**
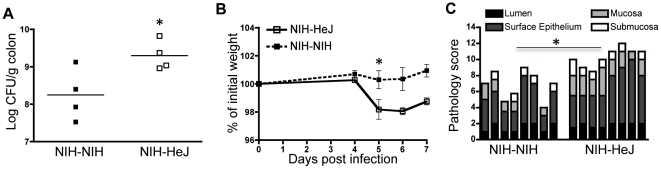
Increased *C. rodentium* colonization (a), weight loss (b) and intestinal pathology (c) in NIH-HeJ as compared NIH-NIH mice after infection. Pathology was assessed in four regions including the lumen, surface epithelium, mucosa and submucosa. Data is representative of 2 independent experiments. * *P*<0.05 ** *P*<0.01 (n = 8 for each experiment).

To substantiate the specific protective role of the NIH microbiota, we performed the reciprocal experiment, where the microbiota of HeJ mice was transferred to NIH mice and susceptibility to *C. rodentium* infection was assessed. The transfer of the microbiota from HeJ mice to NIH mice was less efficient than the transfer from NIH to HeJ (Fig. 1a). However, compared to NIH-NIH, NIH-HeJ mice were less similar to NIH using Bray-curtis metrics (53.8±1.5 vs 61.7±2.2% similarity (*P*<0.05)), which was associated with a depletion or loss of some NIH specific bacterial phylotypes. Of note, one species of uncultured *Prevotella* (accession JF837919) was detected by T-RFLP in all mice receiving an NIH microbiota but below detection in all mice receiving an HeJ microbiota. In one replicate of the experiments, SFB were not affected in NIH-HeJ mice (0.16±0.04% NIH-HeJ and 0.13±0.02% in NIH-NIH), while they were depleted below detection in the second replicate. Although the transfer of HeJ microbiota to NIH mice did not result in a lethal infection as seen in HeJ mice, NIH-HeJ mice showed an increase (*P*<0.05) in *C. rodentium* disease in all parameters measured, including colonization, weight loss and intestinal pathology when compared to NIH-NIH mice (Fig. 3).

### Protective host defense mechanisms are activated by NIH-specific microbiota

**Figure 4:**
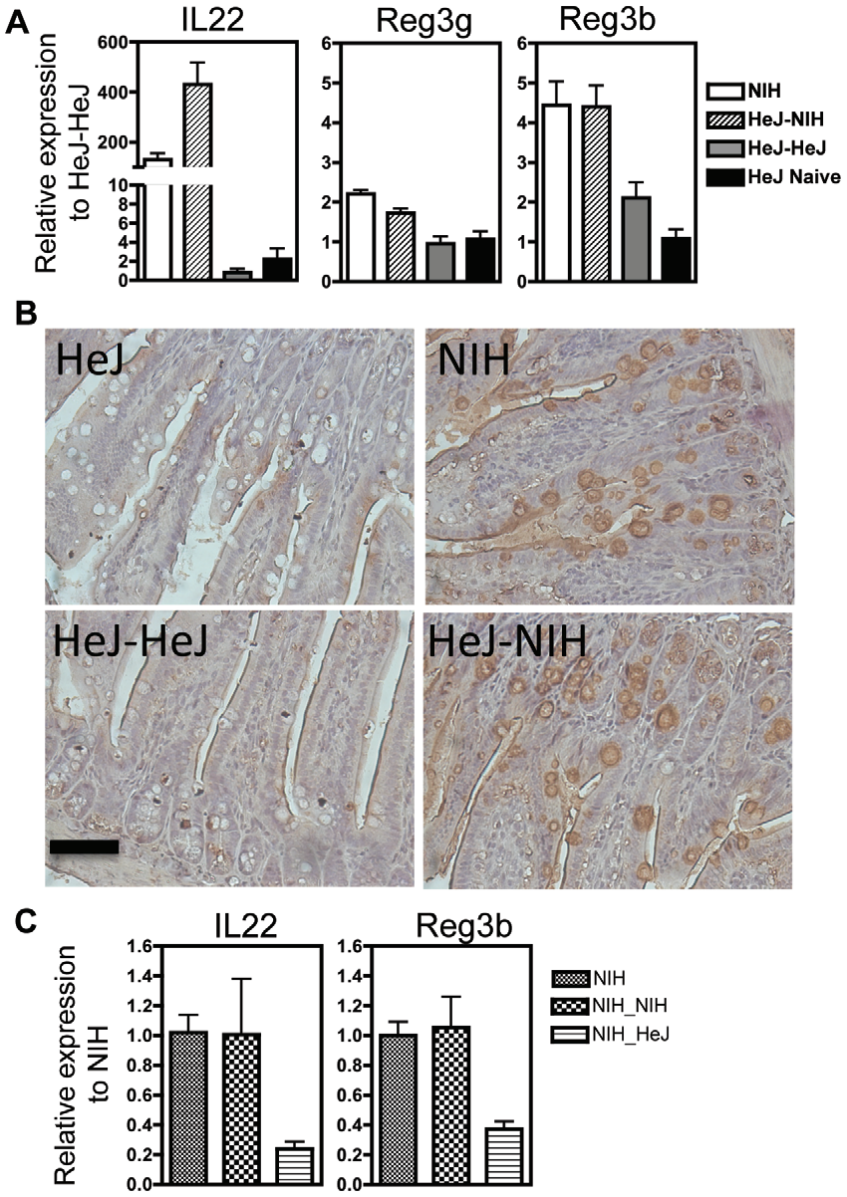
NIH microbiota induced expression of IL-22 and reg3β. Relative expression in the ileum (a) for IL22-, Reg3γ-, and Reg3β-specific mRNA in NIH Swiss (NIH), C3H/HeJ transplanted with NIH microbiota (HeJ-NIH), C3H/HeJ transplanted with HeJ-Naïve (HeJ-HeJ) and C3H/HeJ (HeJ-Naïve) mice relative to HeJ-HeJ mice. (b) Immunostaining for Reg3β in ileal sections shows abundant peptide in ilea of NIH and HeJ-NIH mice, but not in HeJ-HeJ and HeJ-Naïve mice. The scale bar is equal to 50 µm. Data represents mean±SEM. (c) IL-22 and Reg3β expression in NIH mice transplanted with HeJ microbiota (NIH-HeJ) or NIH microbiota (NIH-NIH). All figures representative of two independents experiments with n = 4.

Because of its role in early host defense against attaching and effacing pathogens [20], and the previously demonstrated role of the microbiota in regulating its expression [15], we examined the expression of IL-22 in response to microbial transfer. The NIH microbiota colonizing in HeJ-NIH mice apparently contributed to activation of protective mechanism as shown by increased gene expression of IL-22, mouse Reg3β and Reg3γ in the ileum (Fig. 4a). Reg3β, which has been shown to be protective during Gram-negative bacterial infection in gut mucosa [27], was abundant in the mucosal epithelium of NIH and HeJ-NIH ileum, as detected by immunohistochemistry (Fig. 4b), whereas it was sparse in HeJ-HeJ and HeJ-Naïve mice. Conversely, the depletion of NIH microbiota in NIH-HeJ mice was associated with reduced expression of IL-22 and Reg3β (Fig. 4c), indicating that its expression is dependent on continued stimulation with certain members of the microbiota. The expression of Reg3β was rapidly decreased (over 2-fold within 24 hours) after depletion of NIH microbiota with streptomycin treatment (data not shown).

### Microbially induced protection is IL-22 dependent

**Figure 5:**
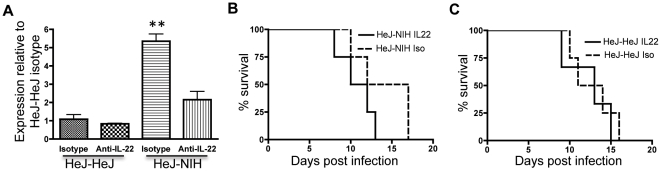
Immunoneutralization of IL-22 impacts survival in HeJ-NIH but not HeJ-HeJ mice. Transcript abundance of Reg3β before infection (a) and survival curves of HeJ mice receiving (b) NIH microbiota or (c) HeJ microbiota after infection with *C. rodentium* and intraperitoneal injection of anti-IL-22 or isotype control. (n = 4).

To determine whether the microbial induction of IL-22 expression prior to infection was important to the observed protection against *C. rodentium* infection we used immunoneutralization of IL-22. Intraperitoneal injection of anti-IL-22 antibodies resulted in reduced Reg3β transcript (Fig. 5a), consistent with the previously demonstrated role of IL-22 in regulating Reg3β expression [20]. Immunoneutralization of IL-22 prior to high-dose infection in HeJ-NIH mice also resulted in earlier mean mortality (11 days) than in mice receiving isotype control (14.5 days) (Fig 5b). Conversely, in HeJ-HeJ mice (Fig. 5c) receiving anti-IL-22 antibodies and isotype control had similar mean mortalities (13 and 12.5 days respectively). Therefore, microbial induction of IL-22 expression and down-stream antimicrobial defenses play a role in the observed microbiota-induced protection against *C. rodentium* infection.

### Protection is not transient

**Figure 6:**
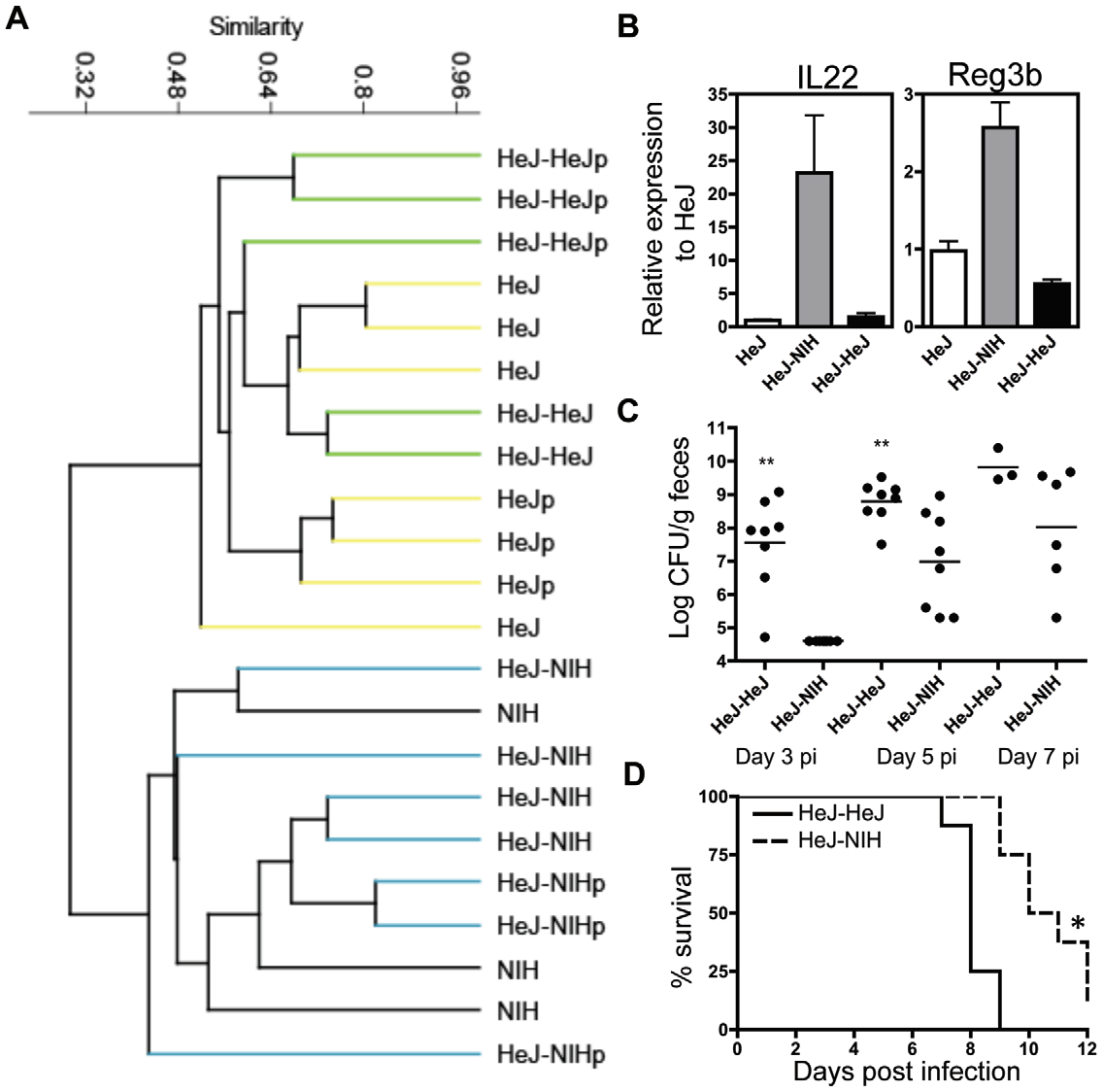
NIH microbiota and improved survival is maintained in the following generation. (a) Similarity tree using Bray Curtis metrics of bacterial 16S rRNA gene terminal restriction fragment profiles from the offspring of untreated C3H/HeJ (HeJ) and NIH Swiss (NIH) mice and HeJ mice having received oral microbial transplantation from HeJ mice (HeJ-HeJ) or NIH mice (HeJ-NIH). Parent mice are identified with a the letter p. (b) Relative expression in the ileum for IL22- and Reg3β-specific mRNA in the offspring of HeJ-Naïve, HeJ-NIH and HeJ-HeJ mice (n = 5). (c) Delayed colonization and (d) mortality in the offspring of HeJ-NIH as compared to HeJ-HeJ mice (n = 8).

To determine whether the effects of microbial transplantation were transient we bred HeJ-NIH and HeJ-HeJ mice 4 weeks after receiving their final microbial transfer. The offspring of the HeJ-NIH, HeJ-HeJ and HeJ mice were then examined as to whether the protective phenotype seen in HeJ-NIH mice was maintained. Indeed, the offspring showed consistent microbial composition and increases in IL-22 and Reg3β expression as their parents (Fig. 6). The offspring of HeJ-NIH mice also showed a corresponding delay in *C. rodentium* colonization on days 3 and 5 after high dose infection and a similar delay in mortality (Fig. 6).

## Discussion

Bacteriotherapy is becoming an increasingly interesting avenue for treatment of intestinal dysfunction. Substantial success has been seen in fecal transplantation to patients suffering from *Clostridium difficile*-associated diarrhea [28]. While this manuscript was in preparation it was also shown that replacing a susceptible C3H/HeOuJ microbiota with a resistant C57BL/6 flora conferred protection against *C. rodentium* induced colitis. This response was correlated with elevated inflammatory responses and improved balance of ion transport after infection, however the pre-infection state was not studied and the responses were all correlative [29]. Despite these advances, this field lacks a clear understanding of mechanisms involved in the transplantation of microbiota.

In this study we show the successful oral transfer of microbiota between hosts of varying susceptibility resulting in protection against *C. rodentium* infection through the induction of host innate defenses, specifically IL-22, demonstrating one mechanism through which microbial transplantation protects against intestinal disease. It has previously been shown that the induction of IL-22 expression upon pathogen colonization is important, as IL-22 ^-/-^ mice are more susceptible to infection [20]. However, here we show that pre-infection levels of expression, which are dependent on the composition of the microbiota, are also important to pathogen success. Expression of the essential cytokine, IL-22, has previously been shown to be mediated by the gut microbiota by increasing intestinal NK46+ lymphocytes [15], although this was demonstrated as a general characteristic of the microbiota. Segmented filamentous bacteria have recently been shown to be highly effective in inducing IL-22 expression [16], and thus the increased colonization of SFB in HeJ-NIH mice likely contributed to the increased expression seen here. However, SFB were not consistently depleted in NIH mice receiving HeJ microbiota, whereas IL-22 expression was consistently reduced. Conversely, the consistent depletion of numerous phylotypes, particularly an uncultured *Prevotella* corresponded with reduced IL-22 expression and increased pathology in NIH-HeJ mice, although this relationship is only correlative. Culture and further characterization of these organisms will be necessary to substantiate a specific IL-22 inducing role.

The mechanism that mediates IL-22 induction remains unclear. The fact that HeJ mice are TLR4^-/-^ rules out the role of TLR4 in the observed response [30]. While TLR4 has been shown to contribute to colitis in response to *C. rodentium* infection, it was also shown to have no effect on host defense during infection [30]. Transfer of NIH microbiota to C3H/HeOuJ mice, which have a functional TLR4 response [30], also increased survival time upon *C. rodentium* infection (data not shown).

The fact that differences in colonization were seen at the early stages of infection supported the role of the innate rather than adaptive immune system in the observed protection. Adaptive immunity is needed for the control and clearance of *C. rodentium* [6], however, innate mucosal defenses including mucin and antimicrobial peptides have been shown to be important for epithelial attachment and luminal colonization [7] [8]. Although increased Reg3β expression correlated with protection we do not explicitly demonstrate its role here. However, the dramatic increase in Reg3β at the protein level observed with immunohistochemistry and the previously demonstrated protective effect of Reg3β against Gram-negative bacterial infection in the gut mucosa [20] [27] would support its role. IL-22 has also been shown to be involved in the development of IL-17-producing T-helper cells [31] and the induction of antimicrobial molecules including Reg3β and Reg3γ [20].

As well as demonstrating a mechanism through which microbial transfer protects the host, this is one of only a few studies demonstrating the feasibility of orally transferring microbiotas between animals of different genetic backgrounds. A recent study using oral transfer of microbiota between rats reported a reduced transfer success when antibiotics were used prior to transfer [32]. However, microbial transfer was only administered once, and at a time point before the antibiotics would have cleared the system. In our experience, antibiotics take a few days to clear (unpublished observation). The highly effective transfer of bacteria between mouse strains would not discount the use of antibiotics in microbial transplantation models.

Consistent with previous reports indicating that microbiota are specific to mouse strain, we found that NIH and HeJ mice had distinct microbial populations, supporting the influence of host genetic determinants in the selection of gut bacteria [33] [34]. However, these mice were from different suppliers, and differences in microbial composition could have been a result of environmental exposure and kinship. That the transferred microbiota was maintained along with its beneficial effects in the next generation of HeJ-NIH mice would support the role of exposure over genetic determinant. However, it has previously been shown that while new bacteria can be introduced to a mouse strain for a generation, clearance can occur through multiple generations as a result of host genotype [35]. Therefore, it is yet unclear whether the absence of protective microbes in HeJ mice was a consequence of host genetics or simply environmental exposure.

That NIH-HeJ mice were not lethally susceptible to infection would suggest that the microbiota composition does not completely account for differences in susceptibility. Indeed, a genetic locus has very recently been identified in C3H mice that makes a marked contribution to susceptibility [36]. It has yet to be determined whether this gene plays a role in shaping microbial composition in C3H mice.

Microbial transplantation provides an excellent model to demonstrate the role of gut microbiota in host defense mechanisms under a similar genetic background. This study demonstrates that the composition of the microbiota can be shifted, resulting in improved host-protection against intestinal infection. Further understanding of how the host acquires a microbiota that beneficially regulates innate defense is still necessary, but indicates that the microbiota contribute extensively to determining individual susceptibility or resistance to infectious agents. To promote the beneficial microbiota for maintenance of good health using bacteriotherapy is therefore becoming an interesting avenue in this field.

## Supporting Information

Figure S1 **Transfer of NIH microbiota resulted in an increased diversity and abundance of Bacteroidetes in HeJ-NIH mice.** Phylogenetic tree of 16S rRNA gene clones from the Bacteroidetes phylum generated from fecal samples from NIH Swiss mice (NIH) (black), C3H/HeJ mice HeJ (Red) and C3H/HeJ mice treated with NIH Swiss microbiota (HeJ-NIH) (blue).
(EPS)

Figure S2 **Transfer of NIH microbiota resulted in reduced Firmicutes in HeJ-NIH mice.** Phylogenetic tree of 16S rRNA gene clones from the Firmicutes phylum generated from fecal samples from NIH Swiss mice (NIH)(black), C3H/HeJ mice (HeJ) (Red) and C3H/HeJ mice treated with NIH Swiss microbiota (HeJ-NIH) (blue).
(EPS)

Figure S3 **Abundance of gut bacteria by qPCR.** Bacterial abundance as assessed by real-time PCR using group specific primers in fecal samples collected from NIH Swiss (NIH), C3H/HeJ transplanted with NIH microbiota (HeJ-NIH), C3H/HeJ transplanted with HeJ-Naïve (HeJ-HeJ), and C3H/HeJ (HeJ-Naïve) on day 14 of microbiota transplantation. Abundance data was corrected relative to eubacteria (all bacteria) and presented as mean±SEM, (n = 6). Data is representative of three independent experiments. * *P*<0.05 ** *P*<0.01.
(TIFF)

Table S1 **Oligonucleotides for real-time PCR and terminal restriction fragment length polymorphism (T-RFLP)**
(DOC)
