# Supplementary figures and images for: Altering Host Resistance to Infections through Microbial Transplantation
Source: PLoS One. 2011 Oct 28;6(10):e26988. doi: 10.1371/journal.pone.0026988 (PMC3203939; doi:10.1371/journal.pone.0026988)

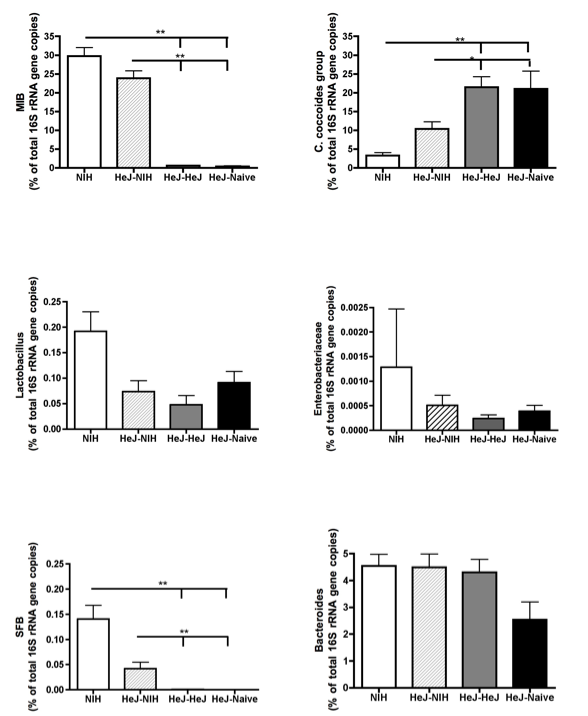

Supplement: Figure S3 — Abundance of gut bacteria by qPCR. Bacterial abundance as assessed by real-time PCR using group specific primers in fecal samples collected from NIH Swiss (NIH), C3H/HeJ transplanted with NIH microbiota (HeJ-NIH), C3H/HeJ transplanted with HeJ-Naïve (HeJ-HeJ), and C3H/HeJ (HeJ-Naïve) on day 14 of microbiota transplantation. Abundance data was corrected relative to eubacteria (all bacteria) and presented as mean±SEM, (n = 6). Data is representative of three independent experiments. * P<0.05 ** P<0.01. (TIFF) [file pone.0026988.s003.tif]
