# Supplementary material for: Altering Host Resistance to Infections through Microbial Transplantation
Source: PLoS One. 2011 Oct 28;6(10):e26988. doi: 10.1371/journal.pone.0026988 (PMC3203939; doi:10.1371/journal.pone.0026988)
Supplement: Table S1 — Oligonucleotides for real-time PCR and terminal restriction fragment length polymorphism (T-RFLP) (DOC) [file pone.0026988.s004.doc]

| **Table S1**. Oligonucleotides for real-time PCR and terminal restriction fragment length polymorphism (T-RFLP) | | |
| --- | --- | --- |
| Target | Sequence (5’-3’) | Annealing  temp (°C) |
| Bacterial 16S rRNA gene (T-RFLP & clones) | F:Fam-AGAGTTTCMTGGCTCAG  R:CCGTCAATTCCTTTRAGTTT | 55 |
| SFB 16S rRNA gene | F:GACGCTGAGGCATGAGAGCAT  R:GACGGCACGGATTGTTATTCA | 58 |
| Clostridium coccoides group | F:ACTCCTACGGGAGGCAGC  R:GCTTCTTAGTCAGGTACCGTCAT | 60 |
| Bacteroides | F:GGTTCTGAGAGGAAGGTCCC  R:GCTGCCTCCCGTAGGAGT | 61 |
| Lactobacilli | F:AGCAGTAGGGAATCTTCCA  R:CACCGCTACACATGGAG | 56 |
| Enterobacteriaceae | F:GTGCCAGCMGCCGCGGTAA  R:GCCATAACGTTGAAAGATGG | 61 |
| MIB | F:CCAGCAGCCGCGGTAATA  R:CGCATTCCGCCTACTTCTC | 58 |
| Eubacteria | F:ACTCCTACGGGAGGCAGCAGT  R:ATTACCGCGGCTGCTGGC | 63 |
| IL-22 | F:GCAATCAGCTCAGCTCCTGT  R:CGCCTTGATCTCTCCACTCT | 60 |
| RegIIIγ | F:AAGCTTCCTTCCTGTCCTCC  R:TCCACCTCTGTTGGGTTCAT | 60 |
| RegIIIβ | F:GGCTTCATTCTTGTCCTCCA  R:TCCACCTCCATTGGGTTCT | 60 |
| IL-17 | F:CTCTCCACCGCAATGAAGAC  R:AGCTTTCCCTCCGCATTGA | 60 |
| IL-6 | F:GAGGATACCACTCCCAACAGACC  R:AAGTGCATCATCGTTGTTCATACA | 60 |
| GAPDH | F:ATTGTCAGCAATGCATCCTG  R:ATGGACTGTGGTCATGAGCC | 60 |
| TGF**β** | F:TGACGTCACTGGAGTTGTACGG  R:GGTTCATGTCATGGATGGTGC | 60 |
